# Dissipated electroosmotic EMHD hybrid nanofluid flow through the micro-channel

**DOI:** 10.1038/s41598-022-08672-5

**Published:** 2022-03-19

**Authors:** M. Bilal, I. Asghar, M. Ramzan, K. S. Nisar, A.-H Abdel Aty, I. S. Yahia, H. A. S. Ghazwani

**Affiliations:** 1 The University of Lahore, Gujrat Campus, Lahore, Gujrat Pakistan; 2 Department of Computer Science, Bahria University, Islamabad, Pakistan; 3 Department of Mathematics, College of Arts and Sciences, Prince Sattam Bin Abdulaziz University, Wadi Aldawaser, 11991 Saudi Arabia; 4 Department of Physics, College of Sciences, University of Bisha, P.O. Box 344, Bisha, 61922 Saudi Arabia; 5 Physics Department, Faculty of Science, Al-Azhar University, Assiut, 71524 Egypt; 6 Advanced Functional Materials and Optoelectronic Laboratory (AFMOL), Department of Physics, Faculty of Science, King Khalid University, P.O. Box 9004, Abha, Saudi Arabia; 7 Research Center for Advanced Materials Science (RCAMS), King Khalid University, P.O. Box 9004, Abha, 61413 Saudi Arabia; 8 Nanoscience Laboratory for Environmental and Biomedical Applications (NLEBA), Semiconductor Lab., Department of Physics, Faculty of Education, Ain Shams University, Roxy, Cairo, 11757 Egypt; 9 Department of Mechanical Engineering, Faculty of Engineering, Jazan University, Jazan, 45124 Saudi Arabia

**Keywords:** Mathematics and computing, Nanoscience and technology

## Abstract

The main objective of the present study is to explore the effects of electromagnetohydrodynamics electroosmotic flow of hybrid nanofluid through circular cylindrical microchannels. An analysis of hybrid nanofluid consisting of four different nanomaterials i.e., single and multiwall carbon nanotubes, silver, and copper is carried out. Yamada–Ota model is employed for the single and multi wall carbon nanotubes, whereas, Xue model is used for the Silver and Copper hybrid nanofluid for specifying the thermal conductivity. The imposed pressure gradient, electromagnetic field and electroosmosis actuated the fluid flow. The flow of heat transfer and Nusselt number with the account of various effects of Joule heating and viscous dissipation under the circumstances of constant heat flux are discussed graphically. The governing system of equations is molded into a system of coupled, nonlinear ordinary differential equations. The shooting technique is used to extract the numerical solutions of the converted system of equations. Also, the outturn of different parameters like Hartman number, the strength of lateral direction electric field, EDL (electric double layer) electrokinetic width, Joule heating parameters on the temperature, and velocity are investigated. The conversion of simple fluid to hybrid nanofluid has greatly alteration in the present model. It has enhanced the thermal properties of fluid. It is also noted that $SWCNT-MWCNT$ based hybrid nanofluid has most influential impact on Nusselt number, temperature distribution and velocity of the fluid. This attempt is useful for the designing of effectual electromagnetic appliances and exquisite.

## Introduction

The transportation process through microfluidics has gained substantial focus because of their diverse utilizations in widespread fields, such as biochemical engineering, micro-electro-mechanical systems (MEMS), chemical separation devices, drug delivery biochips, thermal control of microelectronic devices, and biomedical peculiar techniques^1–3^. In these microfluidic applications, a conventional pressure-driven flow mechanism was actuated, like in the macroscale flows^4,5^. However, in advanced technologies, due to the diminution of microfluidic device sizes, the pressure-driven flows occur congenital disadvantages including a shortage of specific flow control, power loss due to friction, and weak reconfigurability with microscale devices. Because of these mentioned attributes, an optimized way of fluid flowing through these microfluidic devices should be provided. There are three common propulsion tools for the dynamic fluid flow through microscale devices. These include electromagnetic force^6^, electroosmosis^7^, and pressure gradient^8^. For the study of the uses of microfluidic apparatus, the electroosmotic flows have gained vast concern because of its benefits, such as inadequacy of moving parts, unsophisticated design, effectual redesigning with an electric circuit, and reduced sample dispersion^9^. In such a manner, the electroosmosis activation system can be used as a feasible option for the fluid to move through the micro-channel. For the very first time, Reuss^10^ examined the electroosmotic phenomenon through the poriferous clay. Electroosmosis relates to the origination of fluid flow in interaction with a charged substance by applying an electric field to an electrolyte solution. The activity of numerous potential processes, such as surface group ionization and ion adsorption, leads to a separation of the charge at a fluid-solid link when the surface interacts with the electrolyte solution. The introduction of an external electric field thus results in a net movement of ions, depending on the sign of the charge density, to the cathode or anode. Due to viscous drag along the surface, the passage of these ions causes fluid flow. The electroosmotic flow is primarily dependent on the applied electrical fields, the characteristics of the channel wall, and the properties of the electrolyte fluid. Babaie et al.^11^ analyzed the flow of power-law electroosmotic fluid through a micro-cylinder having a slip mechanism with a pressure gradient. They solved the momentum equation numerically followed by the Poisson-Boltzmann equation for both adverse and favorable pressure gradients. Through the circular microchannel, Sun et al.^12^ studied the electroosmotic flow of thermally fully developed power-law non-Newtonian fluid numerically. The energy and Navier Stokes equations are developed by characterizing the Joule heating and Poisson Boltzmann equation for the electroosmotic flow. The electroosmotic incompressible magnetohydrodynamics flow of Maxwell fluid using the separation of variables is investigated by Liu et al.^13^. Lorentz and electric forces are exploited for the driving of the fluid. Ganguly et al.^14^ delineated the thermally developed nanofluid flow through the constant wall temperature micro-channel using the semi-analytical technique. The electroosmotic and pressure-driven flow along with the magnetic field, viscous dissipation, and Joule heating is studied by them for the complex microscale transport processes. Over the parallel slit micro-channel, the pressure-driven electroosmotic unsteady flow with the vertical magnetic field is analytically investigated by Jian^15^. Laplace transform method is utilized for solving the energy equation which comprises volumetric heat generation and viscous dissipation. Within the elliptic and circular microchannels, the electroosmotic power-law model is discussed by Srinivas^16^ in the absence of Debye-Huckel approximations. They found that the flow rate is reduced in the elliptic channel when compared with the circular channel. Xie et al.^17^ offered an overview of electroosmotic flow across the cylindrical micro-channel with magnetohydrodynamic and enforced pressure gradient. Assuming the fixed wall heat flux, the viscous dissipation and Joule heating impacts were investigated graphically.

Electromagnetohydrodynamics has achieved great concern due to a large number of its applications in microfluidics. It is very essential because of its use in different micro-devices such as forcing fluid flow through nano-dimensional channels, biochemical fields, in the fabrication process, two-dimensional control mechanism, and for controlling the electrolyte flows. The Electromagnetohydrodynamics mechanism is also used to control the flow of fluids through the imposing electric fields. In electromagnetohydrodynamics, the forcing force, i.e., the Lorentz force is introduced in the electrically conducting fluid which is greatly affected by the orthogonal magnetic field. In the field of EMHD, Jang and Lee^18^ gave the conception of electromagnetohydrodynamics micropump for measuring the pressure difference and flow rate. Lemoff and Lee^19^ build a practical model of an electromagnetohydrodynamics pump in which the forcing electric force was used to actuate an electrolyte solution through a micro-channel. The participation of electromagnetic force for the surface channel between the parallel plates has been studied by Tso and Sundaravadivelu^20^. The numerical study of laminar flow for an electromagnetohydrodynamics pump has been done by Wang et al.^21^. Abdullah and Duwairi^22^ has analyzed the electromagnetohydrodynamics flow in an alternating current micropump. The flow behaviors due to imposed electromagnetohydrodynamics field together with the electrokinetics effects through microfluidic nanofluids for the heat transfer have been investigated by Rajib^23^. The resisting behavior of the combined electromagnetohydrodynamics field and the pressure-driven force inside the micropumps is analyzed by Shojaeian and Shojaee^24^. Bhatti and Rashidi^25^ combinely portrayed the effects of both electromagnetohydrodynamics and thermal radiation on Riga plate for an irrotational and incompressible viscous nanofluid theoretically. The electrically conducting nanofluid flowing over a Riga plate under the effects of electromagnetohydrodynamics is studied by Ayub et al.^26^. The electromagnetohydrodynamics flow through the curved rectangular micro-channel for the Newtonian fluid is discussed by Liu^27^. Additionally, an entropy generation phenomenon is also studied by him. Qia and Wu^28^ analytically find the solution of electromagnetohydrodynamics flow through a micro-channel in the form of eigenfunction expansion.

One of the advanced ways of improving the heat transfer in the fluids was invented in 1995 after the work of Choi^29^. According to his theory, a mixture named nanofluid is obtained by adding nanoparticles in a small amount to the base or carrier fluid. The nanofluids increase the thermal conductivity of the base fluids which results in a significant effect on the performance of heat transfer. The next phase in this domain is the invention of hybrid nanofluids which has recently magnetized the attention of researchers due to their remarkable features, such as, rate of heat transfer, low cost, electrical and thermal conductivity. Inserting two or more different types of nanoparticles into the base fluids produces hybrid nanofluids. Hybrid nanofluids improve the materials’ physical and chemical properties at the same time. As compared to simple nanofluids^30–33^, the hybrid nanofluids^34–39^ have more effect on the betterment of heat transfer because they have more thermal conductivity and can be molded according to the requirement. Synthetic hybrid nanofluids demonstrate unusual properties that are not presented by individual components. The properties of those composites have been investigated by Li et al.^40^. The amount of difference between the experimental data and the thermal and physical properties of the nanofluids in heat transfer phenomena has been studied by Duangthongsuk and Wongwises^41^. The numerical study of water-based hybrid nanofluid flows through the semicircular region and in the absence of friction force has been done by Chamka et al.^42^. Sheremet and Pop^43^ computationally scrutinized the mixed convection hybrid nano-suspension fluid in moving lid-driven having cooled/heated chamber. They used the finite difference technique for the solution purpose. Abbas et al.^44^ focused on the hybrid nanofluid, flowing over the moving cylinder, having stagnation point flow with an inclined magnetic field. Under specific speculations, they used the prolonged Yamada-Oda and Xue models for the hybrid nanofluid. Concentrating on Single and Multiwall carbon nanotubes and Ethylene glycol as hybrid nanofluid, Shafiq and Nadeem^45^ numerically analyzed the suspension of dust particles using a micropolar fluid with nonlinear thermal radiation and viscous dissipation. Geridonmez and Oztop^46^ numerically studied the hybrid nanofluid having an evenly distributed magnetic field in a backward-facing step channel with mixed convection. Further, they adopted vorticity and stream function for the formulation of governing equations. Over a moving surface, the heat transfer characteristics are numerically investigated by Khashiaie^47^ using the Aluminia-copper/water hybrid nanofluid with melting heat phenomenon. Hanif et al.^48^ explored the magneto-hybrid nanofluid (ferrous oxide-copper/water) having variable viscosity and heat flux through a vertical cone with heat generation and radiation effects. With the Thomas algorithm, the Crank-Nicolson scheme is implemented in MATLAB. Goudarzi et al.^49^ critically viewed the natural convection of Brownian motion and thermophoresis impact on nanoparticles of hybrid nanofluid flowing in sinusoidal wavy insulated walls. Sarkar et al.^50^ summarized the current research on hybrid nanofluid regarding its synthesis, pressure drop characteristics and heat transfer, thermophysical properties, challenges, and possible applications. They conclude that proper hybridization is needed to make the hybrid nanofluids more useful for heat transfer enhancement.

This article admits the magnetohydrodynamic electroosmotic flow of hybrid nanofluid through circular cylindrical micro-channel along with the conduct of imposed pressure gradient and additional electric fields. This article is basically the extension work of Xie et al.^17^. We have studied the impact of hybrid nanofluid on the velocity and temperature of the fluid flowing through micro-channel. The single wall and multi-wall carbon nano-particles and the nanoparticles of silver and copper are infixed to convert the simple fluid into a hybrid nanofluid. Carbon nanotubes are most efficient nanoparticles of cylindrical shape having size 0.5-1.5nm diameter. These nanotubes have six times greater thermal and physicochemical properties as compared to other metallic or non-metallic nanoparticles. CNT are commonly used in micro-electronic cooling purpose, material science, chemical production and in the field of optics. Getting inspired from the advanced research on hybrid nanotechnology, especially on thermal properties of electroosmotic flow through parallel microchannel, a mathematical model is established in this study. The calculated results may be used in many thermal systems such as cooling of microchip devices efficiently^51^, development of improved thermo-chips/micropumping, or microfluidic reactors^52^. Further, we have made a comparative study for the different combinations of hybrid nanofluids. The governing partial differential equations are reborn into dimension-free ordinary differential equations using appropriate similarity transformation. Furthermore, the shooting method is applied to receive numerical results. Eventually, the conduct of velocity and temperature profiles are taken into account. The impact of different parameters on velocity and temperature distribution is encountered through graphs. No such study with hybrid nanofluid is discussed in the literature yet.

## Mathematical representation of the model

**Figure 1:**
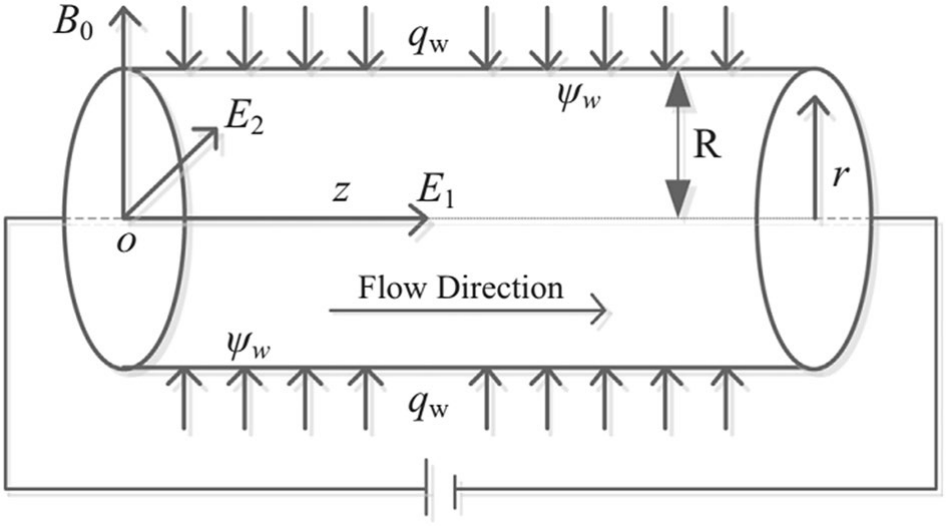
Diagram of flow model.

The transport properties of fully thermally conducted electromagnetohydrodynamics flow because of electroosmosis and imposed pressure gradient through circular cylindrical micro-channel have been considered. A circular cylindrical micro-channel with radius *R* and length *L* is taken into account along the horizontal direction. The length *L* is much greater than the diameter of the cylindrical micro-channel $(L>>R)$. The imposed pressure gradient and externally imposed electric field $E_{1}$ are applied along the principal axis of the cylindrical micro-channel. Another electric field $E_{2}$ from outside to the inside of the micro-channel has been applied in the lateral direction. A uniform magnetic force namely $B_{0}$ is applied in the direction which is perpendicular to the direction of the fluid flow, as shown in Fig. 1. Further, a system of cylindrical coordinates is naturalized at the center of the micro-channel. Also, the simple fluid is changed into the hybrid nanofluid by inserting the nanoparticles of silver, copper, and single and multi-wall carbon nanoparticles. The thermo-physical properties of these particles are given in Table 1. With this modification, the governing momentum equation, and temperature equations are mediocre.

The cylindrical circular microchannel contains the symmetric electrolyte solution. The magnetic field is applied to this solution which may result a deformation in it. When the walls of the micro-channel meet with the electrolyte solution, the surface charge is produced. In this practice, the free ions got separated into the solution. This ionic phenomenon for the evaluation of the net charge density of local volume is presented through the Boltzmann distribution. So, the Poisson Boltzmann equation^17^ exhibiting the phenomenon of electric potential is given by

1 $$\begin{aligned} \nabla ^{2}\psi =\frac{-\rho _{e}}{\epsilon }. \end{aligned}$$

According to previous work done by the researchers in this era^16,17^, the electric potential is a function of radial direction. We apply the Debye Huckel approximation when the thermal potential is more than the electric potential. Then, the resulting Poisson Boltzmann equation becomes^17^,

2 $$\begin{aligned} \frac{1}{r}\frac{\partial }{\partial r}\left( r\frac{\partial \psi }{ \partial r}\right) =\kappa ^{2}\psi , \end{aligned}$$

with

3 $$\begin{aligned} \text {at }r= & {} R,\ \psi =\psi _{w}, \end{aligned}$$

4 $$\begin{aligned} \text {at }r= & {} 0,\ \frac{d\psi }{dr}=0, \end{aligned}$$

where $\kappa$ denotes the Debye Huckel parameter defined as $\left( 2e_{0}^{2}Z_{v}^{2}n_{0}/\epsilon \kappa _{b}T_{a}\right) ^{1/2}$. For the circular cylindrical micro-channel, the volumetric net charge density $\rho _{e}$, therefore becomes as^17^

5 $$\begin{aligned} \rho _{e}=-\epsilon \kappa ^{2}\psi _{w}\frac{I_{0}\kappa r}{I_{0}\kappa R}, \end{aligned}$$

here, the revised Bessel functions $I_{0}$ is of order zero of the first kind. We analyze that the ionic distribution of the solution is moved when the magnetic field is applied to the solution. Consequently, this reflection affects the electric potential in this fluid model. But, we have neglected the influence of the magnetic field on the electric double layer. This results that the imposed electric field is larger than the induced electric field, which is due to the magnetic field. We have considered the magnetohydrodynamic flow through a cylindrical circular micro-channel of an incompressible viscous electrolyte solution. Generally, the fluid slowly moves in micro-fluidic devices in nature which consequences a low Reynolds number. Therefore, the flow is unidirectional. Also, we have considered the symmetrical flow so its velocity is neglected along the angular and radial direction. So, the velocity is only along the $z-$direction. Hence the resulting Naiver’s stokes equation is given by^17^,

6 $$\begin{aligned} \frac{-\partial p}{\partial z}+\frac{1}{r}\frac{\partial }{\partial r}\left( r\mu _{hnf} \frac{\partial v_{z}}{\partial r}\right) +F_{z}=0, \end{aligned}$$

where $v_{z}$ is the velocity along the flow direction, $\mu _{hnf}$ the dynamic viscosity of hybrid nanofluid, *p* the pressure, $$F_{z}\ $$ the electromagnetic body force acting on the fluid along the flow direction. The Lorentz force and electrokinetic effect due to electric and magnetic forces are^17^

7 $$\begin{aligned} \mathbf {F=\rho }_{e}\mathbf {E+J\times B}, \end{aligned}$$

here, **E** is the applied electric field, $\mathbf {J=\sigma _{hnf} } \left( \mathbf {E+u\times B}\right)$ the current density vector, **u** the flow velocity vector, $$\sigma \ $$ the electrical conductivity of the hybrid nanofluid, and **B** is the applied magnetic field. In formal micro-channel flows, the order of magnetic Reynolds number is taken to be $10^{-5}.$ So the induced magnetic field is ignored under the assumption of unidirectional flow and imposed electric and magnetic field, $F_{z}$ becomes^17^

8 $$\begin{aligned} F_{z}=\rho _{e}E_{1}+\sigma _{hnf} B_{0}E_{2}-\sigma _{hnf} B_{0}^{2}v_{z}. \end{aligned}$$

Substituting the values of $F_{z}$ from the above equation into the Naiver’s stokes equation, the resulting momentum equation in the cylindrical coordinate system is given by^17^

9 $$\begin{aligned} \frac{-\partial p}{\partial z}+\frac{1}{r}\frac{\partial }{\partial r}\left( r\mu _{hnf}\frac{\partial v_{z}}{\partial r}\right) +\rho _{e}E_{1}+\sigma _{hnf}B_{0}E_{2}-\sigma _{hnf}B_{0}^{2}v_{z}=0. \end{aligned}$$

The associated boundary conditions of the above equation (9) are^17^,

10 $$\begin{aligned} \left. \begin{aligned}&at\ \ r = R,\ \ \ \ \ \ \ v_{z}=0,\\&at\ \ r = 0,\ \ \ \ \ \ \ \frac{\partial v_{z}}{\partial r}=0.\\ \end{aligned} \right\} \end{aligned}$$

Due to the velocity distribution of magnetohydrodynamic electroosmotic flow, the constant heat wall flux $q_{w}$ basis the thermal transport phenomenon of fluid flow in cylindrical micro-channel. The axial conduction, Joule heating effects, and viscous dissipation factors are applied to the energy equation in a cylindrical coordinate system which results in the differential equation as^17^,

11 $$\begin{aligned} \left( \rho C_{p}\right) _{hnf}v_{z}\frac{\partial T}{\partial z} =k_{hnf}\left( \frac{1}{r}\frac{\partial }{\partial r}\left( r\frac{\partial T}{\partial r}\right) +\frac{\partial ^{2}T}{\partial z^{2}}\right) +\mu _{hnf} \left( \frac{\partial v_{z}}{\partial r}\right) ^{2}+S_{j}, \end{aligned}$$

where $(C_{p})_{hnf}$ is the specific heat of the fluid at constant pressure, $k_{hnf}$ is the thermal conductivity of the hybrid nanofluid, and *T* is the local temperature of the fluid. Here, $S_{j}$ is the volumetric Joule heating generation with $S_{j}=\sigma _{hnf} \left( E_{1}^{2}+E_{2}^{2}\right)$. The Joule heating factor is affected by the weak convective impact. Under the assumption of the larger value of the magnetic field, fully developed thermal flow, and constant heat wall flux, the governing differential equation is^17^

12 $$\begin{aligned} k_{hnf}\left( \frac{1}{r}\frac{\partial }{\partial r}\left( r\frac{\partial T }{\partial r}\right) \right) =\left( \rho C_{p}\right) _{hnf} \left( v_{z} \frac{dT_{m}}{dz}\right) -\mu _{hnf} \left( \frac{dv_{z}}{dz}\right) ^{2}-\sigma _{hnf} \left( E_{1}^{2}+E_{2}^{2}\right) , \end{aligned}$$

here, $v_{z}$ is the velocity along the $z-$direction, $\theta$ is the temperature distribution, *p* the pressure, $\rho _{e}$ the local volumetric net charge density, $T_{w}$ the local wall temperature, $T_{m}$ the bulk temperature.

The associated boundary conditions with the temperature equation are

13 $$\begin{aligned} \left. \begin{aligned}&at\ \ r = R,\ \ \ \ \ \ \frac{dT}{dr}=0 \\&at\ \ r = 0, \ \ \ or\ \ T=T_{w}. \end{aligned} \right\} \end{aligned}$$

The momentum (9) and energy equations (12) can be converted into dimensionless differential equations using the following similarity transformation^17^,

14 $$\begin{aligned} \left. \begin{aligned} & r^{*}=\frac{r}{R}, \quad u^{*}=\frac{\ v_{z}}{u_{e0}}, \quad U_{eo}=\frac{-\varepsilon E_{1}\psi _{w}}{\mu _{f}}, \quad T=\frac{q_{w}R}{k_{f}}\theta +T_{w}, \\ & \psi ^{*} =\frac{\psi }{\psi _{w}}, \quad \tau ^{*}_{1}=\frac{-1}{\tau }\frac{\partial p}{\partial z}, \quad \tau =\frac{\mu _{f}U_{eo}}{R^{2}} \\ & K =k_{f}R, \quad S=\frac{E_{2}R}{U_{eo}}\sqrt{\frac{\sigma _{f}}{\mu _{f}}}, \quad Ha=B_{0}R\sqrt{\frac{\sigma _{f}}{\mu _{f}}}, \end{aligned} \right\} \end{aligned}$$

where, $U_{eo}$ is the reference Helmholtz-Smoluchowski electroosmotic velocity, *K* the electric double layer electrokinetic width, *S* the dimensionless parameter denoting the strength of the lateral direction of electric field and *Ha* are the Hartman number. Also, the volumetric net charge density $\rho _{e}$ and external electric field $E_{1}$ are given by

15 $$\begin{aligned} \rho _{e}=-\epsilon \kappa ^{2}\psi _{w}\frac{I_{0}\left( \kappa r\right) }{ I_{0}\left( \kappa R\right) },\ \ E_{1}=-\frac{\mu _{f}U_{eo}}{\epsilon \psi _{w}}. \end{aligned}$$

The non-dimensional form, after removing the $^{*}$ signs, becomes,

16 $$\begin{aligned}&\tau _{1} +\frac{A_{1}}{r}\frac{\partial }{\partial r}\left( r\frac{\partial u}{ \partial r}\right) +K^{2}\psi +A_{3}S\left( Ha\right) -A_{3}\left( Ha\right) ^{2}u=0.\end{aligned}$$

17 $$\begin{aligned}&\left( \frac{1}{r}\frac{\partial }{\partial r}\left( r\frac{\partial \theta }{\partial r}\right) \right) =\frac{u}{\beta _{1}}\frac{A_{2}}{A_{4}} +\left( \frac{S_{1}+S_{2}}{2}\right) \frac{A_{2}}{A_{4}}\frac{u}{\beta _{1}}+ \frac{A_{2}}{A_{4}}\left( Br\right) \left( \frac{\beta _{2}}{\beta _{1}} \right) u \nonumber \\&\quad -Br\frac{A_{1}}{A_{4}}\left( \frac{du}{dr}\right) ^{2}-\left( S_{1}+S_{2}\right) \frac{A_{3}}{A_{4}}, \end{aligned}$$

The boundary conditions associated with the problem will become as

18 $$\begin{aligned} \left. \begin{aligned}&\text {at}\ r =1,\ \ u=0,\ \ \frac{d\theta }{dr}=1, \\&\text {at}\ r =0,\ \ \frac{\partial u}{\partial r}=0, \ \ \theta = 0, \end{aligned} \right\} \end{aligned}$$

The following non-dimensional number are also useful during the conversion purpose,

19 $$\begin{aligned} \left. \begin{aligned}&\frac{dT_{m}}{dz}=\frac{2\pi Rq_{w}+\sigma _{f}\left( E_{1}^{2}+E_{2}^{2}\right) \pi R^{2}+2\pi \mu _{f} \int \limits _{0}^{R}\left( \frac{dv_{z}}{dr}\right) ^{2}rdr}{\left( \rho C_{p}\right) _{f}v_{m}\pi R^{2}}, \\&v_{m}=\frac{\int \limits _{0}^{2\pi }\int \limits _{0}^{R}v_{z}rdrd\theta }{\pi R^{2}},\ \ \beta _{1}=\int \limits _{0}^{1}u^{*}r^{*},\ S_{1}=\frac{R\sigma _{f} E_{1}^{2}}{q_{w}},\ \ S_{2}=\frac{R\sigma _{f} E_{2}^{2}}{q_{w}}, \\&B_{r}=\left( \frac{\mu _{f} U_{eo}^{2}}{q_{w}R}\right) , \beta _{2}=\int \limits _{0}^{1}\left( \frac{du^{*}}{dr^{*}}\right) ^{2}r^{*}dr^{*}, \end{aligned} \right\} \end{aligned}$$

The physical properties of hybrid nanofluid are defined as^34^;

20 $$\begin{aligned} \left. \begin{aligned}&\mu _{hnf}=\mu _{f}\left( 1-\phi _{1}\right) ^{-\frac{5}{2}}\left( 1-\phi _{2}\right) ^{-\frac{5}{2}}=\mu _{f}A_{1} \\&\left( \rho C_{p}\right) _{hnf}=\left[ \left( 1-\phi _{2}\right) \left( \left( 1-\phi _{1}\right) \left( \rho C_{p}\right) _{f}+\phi _{1}\left( \rho C_{p}\right) _{s_{1}}\right) \right] +\phi _{2}\left( \rho C_{p}\right) _{s_{2}} \\&=\left( \rho C_{p}\right) _{f}\left[ \left( 1-\phi _{2}\right) \left( \left( 1-\phi _{1}\right) +\frac{\phi _{1}\left( \rho C_{p}\right) _{s_{1}}}{ \left( \rho C_{p}\right) _{f}}\right) +\frac{\phi _{2}\left( \rho C_{p}\right) _{s_{2}}}{\left( \rho C_{p}\right) _{f}}\right] =\left( \rho C_{p}\right) _{f}A_{2} \\&\sigma _{hnf}=\left[ \frac{\sigma _{s_{2}}+2\sigma _{bf}-2\phi _{2}\left( \sigma _{bf}-\sigma _{s_{2}}\right) }{\sigma _{s_{2}}+2\sigma _{bf}+\phi _{2}\left( \sigma _{bf}-\sigma _{s_{1}}\right) }\times \frac{\sigma _{s_{1}}+2\sigma _{f}-2\phi _{1}\left( \sigma _{f}-\sigma _{s_{1}}\right) }{ \sigma _{s_{1}}+2\sigma _{f}+\phi _{1}\left( \sigma _{f}-\sigma _{s_{1}}\right) }\right] \\&\sigma _{hnf}=\sigma _{f}A_{3}, \end{aligned} \right\} \end{aligned}$$

The thermal conductivity for the Yamada–Ota model is

21 $$\begin{aligned}&k_{hnf}=k_{bf}\left( \frac{\left( 1+\frac{k_{bf}}{k_{s2}}\frac{L}{R}\phi _{2}^{0.2}\right) +\left( 1-\frac{k_{bf}}{k_{s2}}\right) \frac{L}{R}\phi _{2}^{0.2}+2\phi _{2}\left( \frac{k_{s2}}{k_{s2}-k_{bf}}\right) \ln \left( \frac{k_{s2}+k_{bf}}{k_{s2}}\right) }{\left( 1-\phi _{2}\right) +2\phi _{2}\left( \frac{k_{bf}}{k_{s2}-k_{bf}}\right) \ln \left( \frac{k_{s2}+k_{bf} }{k_{bf}}\right) }\right) , \nonumber \\&k_{bf} =k_{f}\left( \frac{\left( 1+\frac{k_{f}}{k_{s1}}\frac{L}{R}\phi _{1}^{0.2}\right) +\left( 1-\frac{k_{f}}{k_{s1}}\right) \frac{L}{R}\phi _{1}^{0.2}+2\phi _{1}\left( \frac{k_{s1}}{k_{s1}-k_{f}}\right) \ln \left( \frac{k_{s1}+k_{f}}{k_{s1}}\right) }{\left( 1-\phi _{1}\right) +2\phi _{1}\left( \frac{k_{f}}{k_{s1}-k_{f}}\right) \ln \left( \frac{k_{s1}+k_{f}}{ k_{f}}\right) }\right) =k_{f}A_{4}, \end{aligned}$$

and for the Xue model,

22 $$\begin{aligned} k_{hnf} &= k_{bf}\frac{1-\phi _{2}+2\phi _{2}\left( \frac{k_{s1}}{ k_{s1}-k_{bf}}\right) \ln \left( \frac{k_{s1}+k_{bf}}{k_{bf}}\right) }{ 1-\phi _{2}+2\phi _{2}\left( \frac{k_{bf}}{k_{s1}-k_{bf}}\right) \ln \left( \frac{k_{s1}+k_{bf}}{k_{bf}}\right) },\nonumber \\ k_{bf} &= k_{f}\frac{1-\phi _{1}+2\phi _{1}\left( \frac{k_{s2}}{k_{s2}-k_{f}} \right) \ln \left( \frac{k_{s2}+k_{f}}{k_{f}}\right) }{1-\phi _{1}+2\phi _{1}\left( \frac{k_{f}}{k_{s2}-k_{f}}\right) \ln \left( \frac{k_{s2}+k_{f}}{ k_{f}}\right) }=k_{f}A_{4}. \end{aligned}$$

**Table 1:** Thermo-physical properties of nanoparticles and base fluid^34,53^.

Material	$H_{2}O(f)$	*SWCNT*	*MWCNT*	Silver Ag	Copper Cu
$\rho (kgm^{-3}$)	997.1	2600	1600	10500	8933
$C_{p}(Jkg^{-1}K^{-1})$	4179	425	796	235	385
k$(Wm^{-1}K^{-1})$	0.613	6600	3000	429	401
$\sigma (sm^{-1})$	5.5$\times$10$^{-6}$	10$^{6}$	10$^{7}$	6.30$\times$10$^{7}$	5.96$\times$10$^{7}$

## Solution procedure

The exact solution of the above system of differential equations is too complicated as these differential equations are nonlinear and coupled. So, we shall use a numerical technique, namely the shooting method based on the Runge-Kutta method of order 4. Firstly, we convert the system of higher-order differential equations into a system of first-order ordinary differential equations. Then, the missing initial conditions are supposed to integrate the differential equations as an initial value problem using the RK-4 method. The missing initial guesses are modified using Newton’s method. Following symbols are used for converting the higher-order to first-order ODEs.

$$\begin{aligned} u&=y_{1},\qquad \frac{\partial u}{\partial r}=y_{2},\qquad \frac{\partial ^{2}u}{\partial r^{2}}=\frac{dy_{2}}{dr}, \\ \theta&=y_{3},\qquad \frac{\partial \theta }{\partial r}=y_{4},\qquad \frac{\partial ^{2}\theta }{\partial r^{2}}=\frac{dy_{4}}{dr} \end{aligned}$$

Now, for utilization of the Newton Raphson method, the missing initial conditions at $r=0$ are set up as $y_{1}=s$ and $y_{3}=t.$ Now, the system of first order initial value problem becomes

$$\begin{aligned}&\frac{dy_{1}}{dr}=y_{2}, \\&\frac{dy_{2}}{dr}+\frac{1}{r}y_{2}-\frac{A_{3}}{A_{1}}(Ha)^{2}y_{1}=-\frac{ A_{3}}{A_{1}}S(Ha)-\frac{1}{A_{1}}K^{2}\psi -\frac{1}{A_{1}}\tau _{1} , \\&\frac{dy_{3}}{dr}=y_{4}, \\&\frac{dy_{4}}{dr}+\frac{1}{r}y_{4}=\left( \frac{A_{2}}{A_{4}}+\left( \frac{ S_{1}+S_{2}}{2}\right) \frac{A_{2}}{A_{4}}+Br\beta _{2}\frac{A_{2}}{A_{4}} \right) \frac{y_{1}}{\beta _{1}}-Br\left( y_{2}\right) ^{2}\frac{A_{1}}{A_{4} }-\left( S_{1}+S_{2}\right) \frac{A_{3}}{A_{4}}, \end{aligned}$$

and the associated initial conditions for the above model are

$$\begin{aligned} y_{1}=s,\quad y_{2}=0,\quad y_{3}=t,\quad y_{4}=0, \end{aligned}$$

Using the Runge-Kutta method of order 4 to solve the above system with initial guesses *s* and *t*. The initial guesses are updated using Newton’s Raphson method. This numerical iterative method is carried out until we meet the tolerance criteria of $10^{-5}$.

**Table 2:** Comparison of numerical results for the temperature distribution.

r/R	0.0	0.1	0.2	0.3	0.4	0.5	0.6	0.7	0.8	0.9	1.0
Zhao et al.^54^	0.5065	0.5035	0.4939	0.4771	0.4517	0.4159	0.3669	0.3019	0.2177	0.1142	0.000
Present	0.5064	0.5033	0.4936	0.4768	0.4514	0.4158	0.3668	0.3019	0.2177	0.1142	0.000

Table 2 shows the comparison of numerical results in a limiting case with previous published article. A good agreement in both columns is noted.

## Results and discussion

In this section, we are interested in exploring the effects of different emerging parameters on velocity and temperature distribution. The results are highlighted with the help of graphs^2–13^ by exhibiting the influence of different parameters like *Ha*, *Br*, *K*, etc on temperature and velocity profiles. All the figures are drawn for the specific fixed values of parameters such as $Ha=0.3,\ S=1.0,\ K=0.4,\ \psi =0.7,\ \tau =0.4, \ Br=0.01, \ \beta _1=0.02,\ \beta _2=0.03, \ S_1=0.5, \ S_2=0.5,\ \phi _1=0.01, \ \phi _2=0.02$.

**Figure 2:**
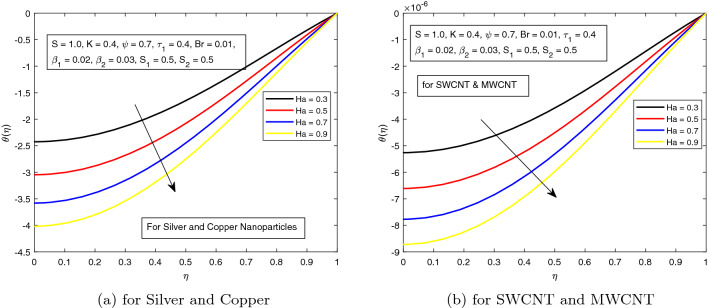
Influence of *Ha* on temperature distribution $\theta ({r})$ for the hybrid nanofluid combination of (a). *Ag* and *Cu*, (b) *SWCNT* and *MWCNT*.

The impact of the Hartman number *Ha* on the temperature distribution is highlighted in Fig. 2. Hartman number denotes the ratio of magnetic forces to viscous forces. In Fig. 2a, b, both combinations of nanoparticles are discussed. It is shown in these figures that temperature declines for higher strength of *Ha*. Usually, higher magnetic parameter, boosts the temperature, but here an inverse observation is noted and it is due to the presence of higher electric field strength which is along the direction of the motion i.e., $(S = 1.0)$. In both combinations i.e. (*a*) Silver and Copper, (*b*) Single and Multiwall CNTs, there is no significant difference for rising temperature. However, the influence of SWNCT and MWCNTs is more prominent than the *Ag* & *Cu* hybrid-nanofluids.

**Figure 3:**
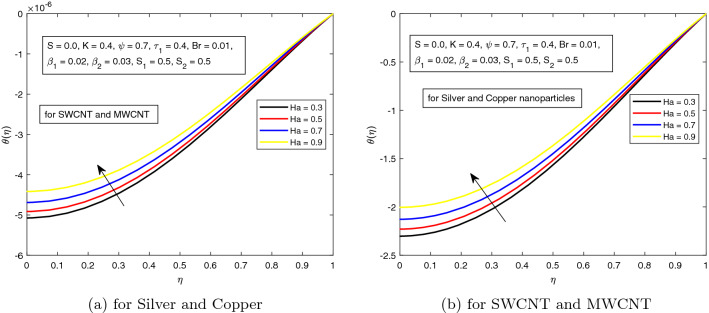
Influence of *Ha* on temperature distribution $\theta ({r})$ for the hybrid nanofluid combination of (a). *Ag* and *Cu*, (b) *SWCNT* and *MWCNT*.

In the next Fig. 3, the variation of Hartman number on the temperature profile, in the absence of electric field is displayed. As expected, and discussed earlier, the temperature of the hybrid nanofluid in the absence of electric field, rises due to higher magnetic parameter. This happens due the higher Lorentz forces which appears due to Hartman number.

**Figure 4:**
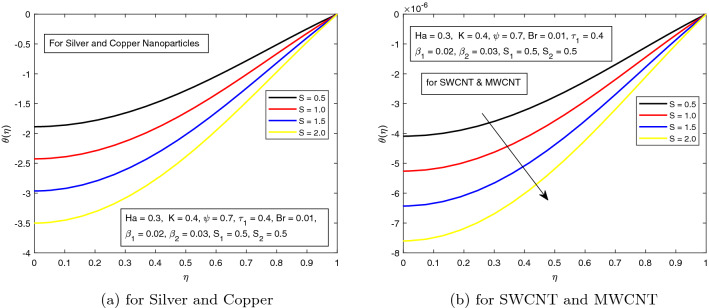
Influence of *S* on temperature distribution $\theta ({r})$ for the hybrid nanofluid combination of (a). *Ag* and *Cu*, (b) *SWCNT* and *MWCNT*.

**Figure 5:**
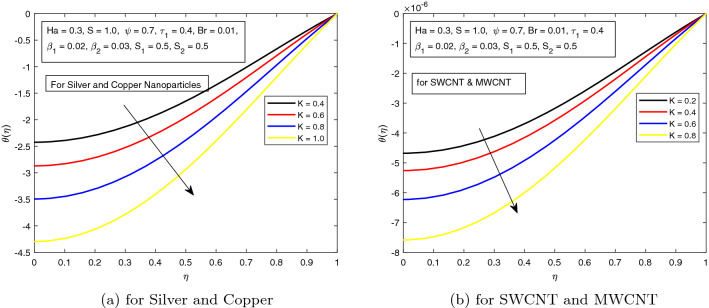
Influence of *K* on temperature distribution $\theta ({r})$ for the hybrid nanofluid combination of (a). *Ag* and *Cu*, (b) *SWCNT* and *MWCNT*.

**Figure 6:**
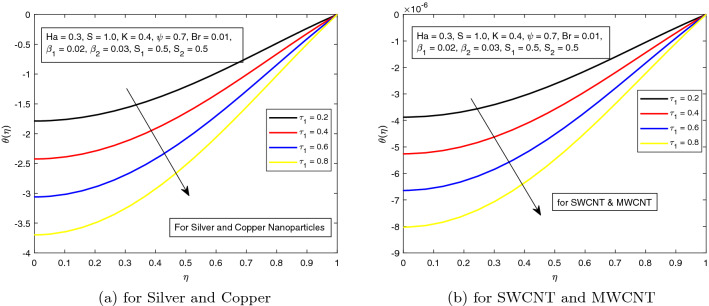
Influence of $\tau _{1}$ on temperature distribution $\theta ({r})$ for the hybrid nanofluid combination of (**a**). *Ag* and *Cu*, (**b**) *SWCNT* and *MWCNT*.

**Figure 7:**
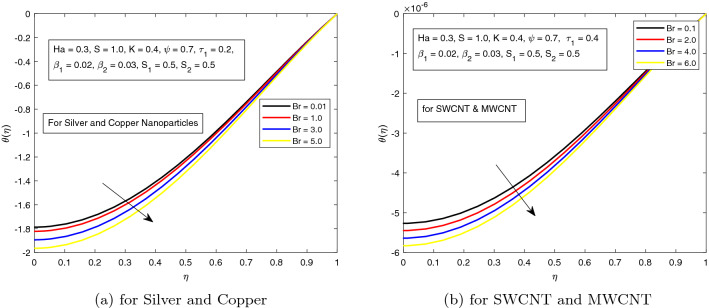
Influence of $\tau _{1}$ on temperature distribution $\theta ({r})$ for the hybrid nanofluid combination of (**a**). *Ag* and *Cu*, (**b**) *SWCNT* and *MWCNT*.

**Figure 8:**
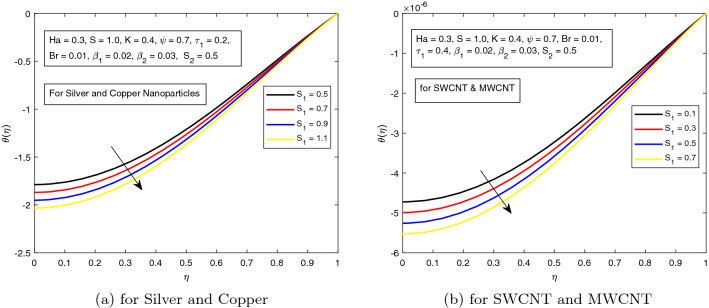
Influence of $S_{1}$ on temperature distribution $\theta ({r})$ for the hybrid nanofluid combination of (**a**). *Ag* and *Cu*, (**b**) *SWCNT* and *MWCNT*.

**Figure 9:**
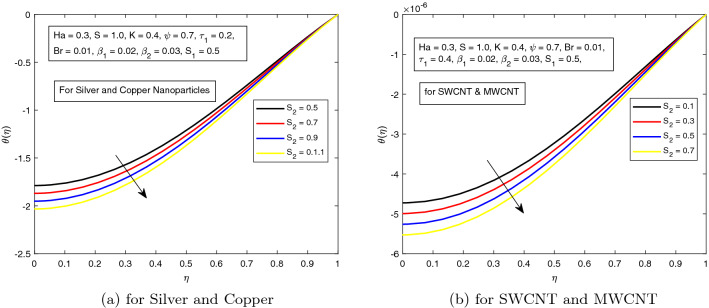
Influence of $S_{2}$ on temperature distribution $\theta ({r})$ for the hybrid nanofluid combination of (**a**). *Ag* and *Cu*, (**b**) *SWCNT* and *MWCNT*.

*S* is a dimensionless parameter that stands for the strength of the lateral direction electric field. Its consequences on temperature profile is exhibited in Fig. 4a. As the electric field is applied in the lateral direction, which allows the fluid to move even faster, and as a result the temperature of the fluid goes down. The hybrid nanofluid consisting of Single and Multi wall carbon nanotubes have more influence on temperature profile. The impact of *K* along the temperature profile has been delighted in Fig. 5. *K* is the electric double layer electrokinetic width corresponding to the ratio of the radius of the base of cylindrical micro-channel to the reciprocal of Debye length $\frac{1}{k}$. It is observed that in both cases i.e. (*a*) Silver and Copper, (*b*) single and multi-wall CNTs, the temperature profile has a falling trend as demonstrated in Fig. 5. The influence of pressure gradient parameter $\tau _{1}$ on temperature distribution is displayed in Fig. 6. It is revealed from the figure that the temperature declines for the increasing value of pressure gradient parameter. This decrement in temperature is due to lowering dynamics viscosity and higher pressure gradient. Again, the decline is more prominent in *SWCNT* and *MWCNT* nanofluid. When the fluid is changed into a hybrid nanofluid by inserting the nanoparticles of different combinations of silver and copper, and single and multi-wall carbon nanotubes, then from Fig. 7 it is noted that increasing values of Brinkman number *Br* decreases the temperature of the fluid. Brinkman number is the ratio of viscous dissipation to the applied heat flux. The viscous dissipation acts like an energy source for the liquid. For larger values of *Br* i.e. for minimum heat flux, the temperature distribution decreases. After performing the careful analysis, it is observed that the combination of multi and single wall CNTs as nanoparticles shows the most decrement in the temperature for the higher *Br*. In the next two figures, the influence of $S_{1}$ and $S_{2}$ on the temperature profile is highlighted. $S_{1}$ and $S_{2}$ are the relative strength of Joule heating to applied heat flux, which can be seen as the dimensionless Joule heating parameters. So, $S_{1}$ and $S_{2}$ are basically dependent on Joule heating parameter. These parameters are defined as $\frac{R \sigma _{f}E_{1}^2}{q_{w}}$ and $\frac{R \sigma _{f}E_{2}^2}{q_{w}}$ respectively. It can be noted that both $S_{1}$ and $S_{2}$ are inversely related to applied heat flux. Heat flux reduces when these parameters are escalates. Due to reduction of heat flux, the temperature of the fluid decreases as demonstrated in Figs. 8 and 9.

**Figure 10:**
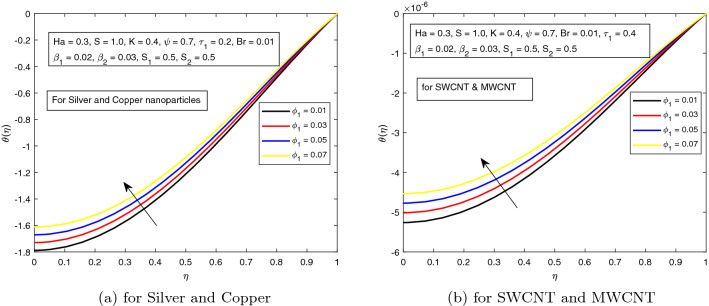
Influence of $\phi _{1}$ on temperature distribution $\theta ({r})$ for the hybrid nanofluid combination of (**a**). *Ag* and *Cu*, (**b**) *SWCNT* and *MWCNT*.

**Figure 11:**
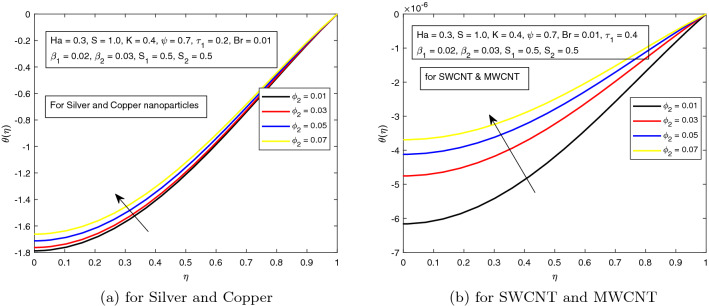
Influence of $\phi _{2}$ on temperature distribution $\theta ({r})$ for the hybrid nanofluid combination of (**a**). *Ag* and *Cu*, (**b**) *SWCNT* and *MWCNT*.

The dimensionless volume fraction of nanoparticles $\phi _{1}$ and $\phi _{2}$ exhibit a prominent role in the energy equation. Their impact on non-dimensional temperature is displayed in Figs. 10a, b and 11a, b. Thermal conductivity and other physical properties of the working fluid are directly related with $\phi _{1}$ and $\phi _{2}$. So, practically, at some instant, the temperature of hybrid nanofluid uplifts by increasing the magnitudes of volume fractions.

**Figure 12:**
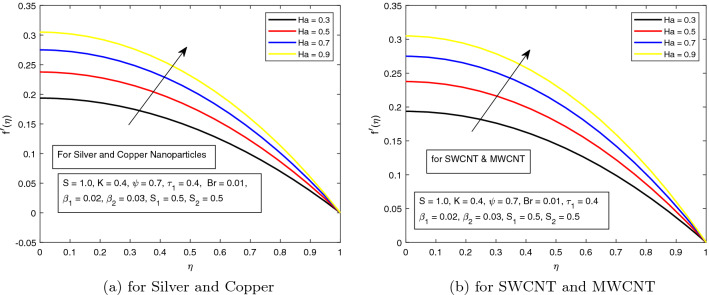
Influence of $\phi _{2}$ on velocity profile $f^{\prime }({r})$ for the hybrid nanofluid combination of (**a**). *Ag* and *Cu*, (**b**) *SWCNT* and *MWCNT*.

In the very next figure, the influence of Hartman number *Ha* on the velocity distribution is foreground. In Fig. 12a, b, both combinations of nanoparticles are hashed out. It is shown in these figures that velocity increases for higher strength of *Ha*. This is due to the presence of electric field which is along the direction of the flow. This electric force dominates the resistive Lorentz forces which act along the direction of flow and distort the flow of the fluid. In both combinations i.e. (*a*) Silver and Copper (*b*) Single and Multiwall CNTs, there is no significant difference for falling velocity. However, again, the influences of SWCNTs and MWCNTs is more prominent.

**Figure 13:**
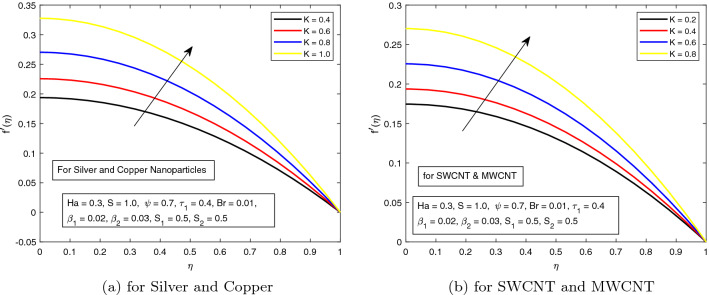
Influence of $\phi _{2}$ on velocity profile $f^{\prime }({r})$ for the hybrid nanofluid combination of (**a**). *Ag* and *Cu*, (**b**) *SWCNT* and *MWCNT*.

The behavior of *K* along velocity distribution is opposite to that of temperature as depicted in Fig. 13a, b. The velocity profile is raising upward by increasing the value of *K*. It is due to the fact that electric double layer electrokinetic width is inversely related to the Debye length.

**Table 3:** Numerical results of Nusselt number when $S_1=S_2=0.5, \beta _1=0.02, \beta _{2}= 0.03, \tau = 0.4$ with boundary condition $\theta ^{\prime }(0)=0$.

*Ha*	*S*	*K*	*Br*	$\phi _{1}$	$\phi _{2}$	$\tau _{1}$	$Cu~ \& ~Ag$	$SWCNT~ \& ~MWCNT$
0.3	1.0	0.4	0.01	0.01	0.02	0.4		
0.4							0.7286004	0.7397778
0.5							0.6566385	0.6666179
0.6							0.6015530	0.6106293
	0.5						1.0603663	1.0773339
	0.7						0.9517140	0.9667369
	0.9						0.8632592	0.8767337
		0.5					0.7619095	0.7736484
		0.6					0.6968497	0.7074959
		0.7					0.6329730	0.6425629
			0.1				0.8235783	0.8363727
			0.5				0.8176499	0.8303616
			1.0				0.8103583	0.8229682
				0.02			0.8415514	0.8579361
				0.04			0.8764943	0.9007912
				0.06			0.9139335	0.9473335
					0.03		0.8395900	0.8578369
					0.05		0.8702421	0.9004450
					0.07		0.9028292	0.9466622
						0.5	0.7291958	0.7403832
						0.6	0.6533756	0.6633011
						0.7	0.5918380	0.6007563

The influence of Hartman number *Ha*, unsteadiness parameter *S*, electrokinetic parameter *K*, Brinkman number *Br* and solid volume fractions $\phi _1$, pressure gradient parameter, and $\phi _2$ on the Nusselt number is displayed in Table 3. The solid volume fraction of both nanoparticles causes the Nusselt number to increase, whereas the unsteadiness parameter, electrokinetic parameter, Hartman number, pressure gradient parameter and Brinkman number show a decreasing trend. An enhancement in the heat transfer rate because of the enhancement of nanoparticles volume fraction is due to the increment in thermal conductivity of the fluid. Higher thermal conductivity boosted the temperature of the fluid on the surface of micro-channel. Nusselt number declines for the increasing Hartman number. Due to higher Lorentz forces, more resistive impact creates in fluid which enhances the temperature of the fluid, and as a result Nusselt number decreases. On the other hand, the Brinkman number is the ratio of viscous dissipation to the applied heat flux. A higher Brinkman number causes a lower heat flux on the wall which resultantly decelerates the rate of heat transfer. From the table, it is clear that the combination of Single and multi wall carbon nanotubes hybrid nanofluid is more effective than the silver and copper hybrid nanofluid.

## Conclusion

The main motive of this work is to study the electroosmotic magnetohydrodynamic flow of a hybrid nanofluid in a circular cylindrical micro-channel. Due to the presence of hybrid nanofluid, the thermal conductivity and other physical properties of current flow are enhanced. Four basic types of nanoparticles i.e., $Cu,\ Ag,\ SWCNT, \ and MWCNT$ are taken into account. Both the electric and magnetic fields along with the pressure gradient are imposed on the flow. The velocity and temperature profiles are examined graphically under the laying claims of constant heat wall flux, one-directional flow in a symmetric angular direction. Due to the influence of various emerging parameters, the conduct of velocity and temperature profiles has been observed with the help of graphs. The main findings are as follows;

- The conversion of simple fluid to hybrid nanofluid has greatly alteration in the present model. It has enhanced the thermal properties of fluid.
- By increasing the volume fractions, the quantity of nano-dimensional particles in the base fluid increases which reveals the change in both temperature and velocity profiles of the flow.
- The increasing Hartman number *Ha* lifted the temperature and velocity of the hybrid nanofluid upward due to resistive forces and applied electric field.
- The Brinkman number *Br* influences the thermal properties of hybrid nanofluid by declining the temperature as we increase it’s magnitudes.
- Numerical results of Nusselt number indicates that $SWCNT-MWCNT-H_2O$ is the more feasible combination of hybrid nanofluid for the rate of heat transfer study as compared to other hybrid nanofluid.
- The electrokinetic parameter which is the ratio of micro-tube radius to the Debye Huckel parameter, has an ascent impact on velocity distribution.

## Data Availability

The datasets used and/or analysed during the current study available from the corresponding author on reasonable request.

## List of symbols

$\mathbf{B }$: Applied magnetic field
$B_{0}$: Uniform magnetic field
$E_{1}$: External electric field
$\rho _{e}$: Local volumetric net charge density
$\psi _{w}$: Uniform wall zeta potentia
$$n_{0}$$: Ion density
$$e_{0}$$: Electron charge
*Nu*: Nusselt number
$$T_{a}$$: Absolute temperature
$\kappa$: Debye–Hückel parameter
*K*: EDL electrokinetic width
$v_{m}$: Axial mean velocity
$v_{\theta }$: Velocity flow in angular direction
*p*: Pressure
*E*: Applied electric field
*R*: Radius of cylinder
(*r*, *z*): Cylindrical axis
$\mathbf{u }$: Flow velocity vector
$q_w$: Constant heat wall flux
$S_1, S_2$: Dimensionless Joule heating parameter
*Br*: Brinkman number
$C_{p}$: Specific heat of the liquid at constant pressure
$E_{2}$: Another electric field
$$\psi$$: Electric potential
$\epsilon$: Permitivity of the medium
$$z_{v}$$: Ion valence
$$k_{b}$$: Boltzmann constant
*T*: Local temperature of the liquid
$$T_{m}$$: Bulk temperature
*k*: Thermal conductivity of the liquid
$F_{z}$: Electromagnetic body force
$v_{r}$: Velocity flow in radial direction
$v_{z}$: Velocity along flow direction
$$\mu$$: Dynamic viscosity
$\sigma$: Electrical conductivity of the liquid medium
*L*: Length of cylinder
$$I_{0}$$: Bessel function
$\mathbf{J }$: Current density vector
$S_j$: Volumetric Joule heating
$\phi _1, \phi _2$: Nanoparticle volume fraction
